# Nutritional composition, bioactive potential, and *in vitro* rumen fermentation of tropical brown *(Sargassum binderi)* and green *(Kappaphycus striatum)* seaweeds as functional feed additives for ruminants

**DOI:** 10.14202/vetworld.2025.3335-3351

**Published:** 2025-11-06

**Authors:** Laras Sukma Sucitra, Mardiati Zain, Fauzia Agustin, Yetti Marlida, Despal Despal, Bella Veliana Utami, Sharli Asmairicen

**Affiliations:** 1 Doctoral Program, Faculty of Animal Science, Universitas Andalas, Padang, West Sumatra, Indonesia; 2 Department of Animal Nutrition, Faculty of Animal Science, Universitas Andalas, Limau Manis Campus, Padang, West Sumatra, Indonesia; 3 Department of Animal Nutrition and Feed Technology, Faculty of Animal Science, IPB University, Bogor, West Java, Indonesia; 4 Research Center for Animal Husbandry, National Research and Innovation Agency (BRIN), Cibinong, Indonesia

**Keywords:** functional feed additive, *Kappaphycus striatum*, methane mitigation, rumen fermentation, *Sargassum binderi*, tropical seaweed

## Abstract

**Background and Aim:**

Mitigating enteric methane emissions in ruminants remains a global challenge in achieving sustainable livestock production. Although seaweed supplementation has shown promising results, most research has focused on temperate species, leaving tropical species underexplored. This study investigated the nutritional composition, bioactive compounds, and *in vitro* rumen fermentation characteristics of two tropical seaweeds, brown seaweed (*Sargassum binderi*) and green seaweed (*Kappaphycus striatum*), as potential functional feed additives for ruminants.

**Materials and Methods:**

The proximate composition, macro- and micro-minerals were determined using Association of Official Analytical Chemists and Inductively Coupled Plasma–Optical Emission Spectrometry methods. *In vitro* digestibility of dry matter digestibility (DMD) and organic matter digestibility (OMD) was evaluated using the Tilley and Terry two-stage technique. Rumen fermentation characteristics, pH, ammonia (NH_3_), and total volatile fatty acids (VFA), were analyzed after 48 h of incubation. Amino acids and fatty acids were profiled using high-performance liquid chromatography and gas chromatography–flame ionization detection, respectively, while bioactive metabolites were identified through liquid chromatography–high-resolution mass spectrometry metabolomics.

**Results:**

Green seaweed exhibited a higher crude protein content (7.52%) and digestibility (DMD = 73.56%; OMD = 72.71%) than brown seaweed (6.84%; 46.38%; 44.99%). VFA production (136.75–151.75 mM) and NH_3_ concentrations (22.21–26.78 mM) differed significantly (p < 0.01) between species, while pH remained within the optimal range (7.00–7.21). Both seaweeds contained balanced essential and non-essential amino acid profiles and abundant polyunsaturated fatty acids, notably linoleic, α-linolenic, eicosapentaenoic acid, docosahexaenoic acid, and conjugated linoleic acid. Metabolomic screening identified ~85 bioactive compounds, including lipid-derived metabolites, amino alcohols, vitamins, and osmolytes such as betaine and cholecalciferol, indicating their potential to modulate rumen fermentation and enhance animal resilience.

**Conclusion:**

Both *S. binderi* and *K. striatum* demonstrated promising nutritional and bioactive potential as ruminant feed additives. Their compositional diversity suggests species-specific applications – *S. binderi* as an energy-dense supplement and *K. striatum* as a functional additive for stress adaptation. However, further *in vivo* trials are necessary to determine optimal inclusion levels, long-term safety, and methane mitigation efficacy under production conditions.

## INTRODUCTION

Greenhouse gas (GHG) emissions from the livestock sector account for approximately 24.5%–29% of total global emissions, a figure comparable to that of the transport industry [1–3]. Among these, methane emissions represent a major concern, not only for their potent greenhouse effect but also because they signify a substantial loss of feed energy that could otherwise support animal growth and productivity [4]. With increasing global attention on climate change and the rising demand for animal-derived food products, there is an urgent need for sustainable innovations to reduce ruminant methane emissions without compromising productivity.

One promising approach is seaweed supplementation, which provides a rich source of bioactive compounds known to suppress enteric methane production [5], enhance nutrient utilization, and improve overall ruminant performance [3, 6–8]. The incorporation of plant-derived bioactives into livestock feed has been shown to modulate rumen microbial activity, improve nitrogen metabolism, and alleviate issues such as bloat [9–13].

As the world’s second-largest seaweed producer, Indonesia possesses abundant tropical marine resources with exceptional biodiversity and favorable growing conditions [14, 15]. However, research efforts on methane mitigation have largely centered on temperate and subtropical seaweeds, particularly the red seaweed *Asparagopsis taxiformis* [16, 17]. While this species demonstrates remarkable methane inhibition, its large-scale use in tropical systems is constrained by concerns regarding sustainability, limited availability, and excessive iodine content. In contrast, tropical seaweed species such as brown seaweed (*Sargassum binderi*) and green seaweed (*Kappaphycus striatum*) remain underexplored despite their abundance and potential relevance to local feed systems. Limited data on their nutritional composition, bioactive profiles, and effects on rumen fermentation hinder their practical adoption as sustainable feed additives in tropical livestock production.

A previous study by Liu *et al*. [18] has reported methane reduction ranging from 66% to 99% for *A. taxiformis* under both *in vitro* and *in vivo* conditions. Brown seaweeds generally yield moderate reductions (<50%), while certain green seaweeds, such as *Cladophora patentiramea*, can achieve up to 66% reduction [17]. Other species, including *Caulerpa taxifolia*, *Cladophora coelothrix*, *Chaetomorpha linum*, *Ulva* spp., and *Ulva ohnoi*, exhibit methane suppression between 27% and 50% after 72 h of incubation [19]. Given the vast and untapped diversity of tropical seaweeds, particularly in Indonesia, further systematic research is essential to identify and optimize native species with high potential for ruminant methane mitigation and sustainable feed innovation [18].

Despite the growing body of evidence supporting the use of seaweeds as sustainable feed additives for mitigating enteric methane emissions in ruminants, most existing research has been restricted to temperate and subtropical species, particularly the red seaweed *A. taxiformis*. Numerous studies have demonstrated its potent antimethanogenic activity; however, its large-scale application in tropical regions remains impractical due to several limitations. These include restricted availability, high production costs, ecological sustainability issues, and excessive iodine and bromoform content, which raise potential toxicity and food safety concerns for livestock and humans. Consequently, there is a lack of research focusing on tropical seaweed species, which are abundant, locally adaptable, and more environmentally sustainable for integration into regional livestock systems such as those in Indonesia.

Furthermore, most previous investigations have emphasized the antimethanogenic efficacy of seaweeds, often neglecting a holistic evaluation of their nutritional value, amino acid and fatty acid composition, mineral content, and the diversity of bioactive metabolites that can influence rumen fermentation and animal productivity. Although *Sargassum* and *Kappaphycus* genera are widely available across Indonesian coastal regions, data describing their nutritional composition, metabolomic profiles, and *in vitro* rumen fermentation responses are virtually absent. This critical information gap limits the scientific understanding and potential formulation of balanced, functional seaweed-based ruminant feeds tailored for tropical production environments.

Thus, a comprehensive characterization of brown (*S. binderi*) and green (*K. striatum*) seaweeds, particularly in terms of their proximate composition, mineral profiles, bioactive compounds, and *in vitro* digestibility and fermentation parameters, is urgently needed. Such studies will help determine their suitability as locally sourced feed ingredients that can enhance nutrient utilization and contribute to enteric methane mitigation strategies in tropical livestock production systems.

This study aimed to evaluate and compare the nutritional composition, bioactive compound profiles, and *in vitro* rumen fermentation characteristics of two tropical seaweed species, *S. binderi* (brown seaweed) and *K. striatum* (green seaweed), collected from Indonesian coastal regions. Specifically, the objectives were to:

1. Determine their proximate, mineral, amino acid, and fatty acid compositions
2. Assess their *in vitro* dry matter (DM) and organic matter (OM) digestibility as indicators of feed utilization potential
3. Evaluate their effects on key rumen fermentation parameters, including pH, ammonia (NH_3_), and total volatile fatty acids (VFA)
4. Identify and characterize bioactive compounds through metabolomic profiling to explore their potential functional roles in methane mitigation and rumen modulation.

By bridging the existing knowledge gap, this study provides the first integrated assessment of these two tropical seaweed species as potential ruminant feed additives, contributing to the development of sustainable, regionally sourced strategies for improving animal productivity and reducing environmental impacts in tropical livestock production systems.

## MATERIALS AND METHODS

### Ethical approval

Ethical clearance was not required for this study as no live animals were used. Rumen fluid was obtained as a byproduct from goats immediately after slaughter for human consumption at a licensed abattoir. This collection method complies with institutional and national ethical guidelines, which exempt the use of abattoir-sourced biological materials from ethical review. The procedure ensured humane sourcing while maintaining biosafety and sample integrity.

### Study period and location

The study was conducted from May 2024 to September 2024 at the Ruminant Nutrition Laboratory, Faculty of Animal Sciences, Universitas Andalas, Padang, West Sumatra, Indonesia; the Dairy Nutrition Laboratory, Faculty of Animal Sciences, Bogor Agricultural Institute, Bogor, West Java, Indonesia; and the Yogyakarta Advanced Characterization Laboratory, National Research and Innovation Agency, Yogyakarta, Indonesia.

### Study design

This research employed a laboratory-based experimental approach using a completely randomized design in a 2 × 4 factorial arrangement, comprising three treatments and four replications for *in vitro* trials. The treatments involved two tropical seaweed species, brown seaweed (*S. binderi*) and green seaweed (*K. striatum*), which were evaluated for their nutritional composition, bioactive compound profiles, and rumen fermentation characteristics under controlled conditions.

### Sampling

Two tropical seaweed species were used: brown seaweed (*S. binderi*) collected from Sungai Nipah, Pesisir Selatan, West Sumatra, and green seaweed (*K. striatum*) collected from Palette Village, East Tanete Riattang Subdistrict, Bone Regency, South Sulawesi, in June 2024. The species were authoritatively identified by Professor Maria Endo Mahata from the Nutrition and Feed Technology Department, Faculty of Animal Science, Universitas Andalas, Padang, Indonesia.

The collected samples were thoroughly washed with freshwater to remove salts, epiphytes, and surface contaminants. Seaweeds were first sun-dried for 48 h, followed by oven-drying at 60°C for another 48 h until a constant weight was achieved. The dried materials were ground using a laboratory mill, then sieved through a 1-mm mesh for chemical composition analysis and a 20-mesh sieve for *in vitro* fermentation assays. The powdered samples were stored in airtight containers at 26.8°C (room temperature) until further analysis.

### Chemical composition analysis

#### Proximate composition

The proximate composition of each seaweed species was determined following Association of Official Analytical Chemists (AOAC) methods [20]. Analyses included the determination of DM, OM, ash, crude protein (CP), and crude fat (CF) contents.

#### Mineral analysis

To assess macro- and micromineral content, samples were oven-dried at 60°C for 24 h, ground, and sieved through a 20-mesh screen to obtain a homogeneous powder. One gram of the sample was suspended in 2 mL of distilled water and heated in a furnace at 150°C for 15 min. After cooling, the sample was diluted to 25 mL with distilled water and filtered through a 45-mesh filter paper. The resulting filtrate was analyzed for macro (calcium [Ca], phosphorus [P], sodium [Na], magnesium [Mg], sulfur [S]) and micro (iron [Fe], manganese [Mn], zinc [Zn], copper [Cu]) minerals using Inductively Coupled Plasma–Optical Emission Spectrometry (ICP-OES; model 5110, Agilent Technologies, Santa Clara, California, USA).

### *In vitro* digestibility and rumen fermentation

*In vitro* fermentation was performed according to the method of Tilley and Terry [21] to evaluate dry matter digestibility (DMD), organic matter digestibility (OMD), and rumen fermentation characteristics after 48 h of incubation. Each treatment was conducted in duplicate, ensuring analytical accuracy and reproducibility.

Rumen fluid was collected immediately post-slaughter from goats at a licensed abattoir. The contents were aseptically aspirated using a syringe, filtered through four layers of cheesecloth into a pre-warmed (39°C) thermos flask, and transported promptly to the laboratory to preserve anaerobic microbial viability. To terminate fermentation, the incubation flasks were immersed in ice water. The pH was recorded immediately using a digital pH meter. The fermentation mixture was centrifuged at 1,372 × *g* for 30 min at 40°C to separate the supernatant and residue.

The supernatant was used to determine the NH_3_ concentration via the Conway microdiffusion method and to measure total VFAs through steam distillation. The residual solids were filtered using Whatman No. 41 filter paper (Cytiva, Marlborough, Massachusetts, USA), oven-dried at 60°C, and then used to calculate DMD and OMD values, which served as indicators of feed digestibility and rumen fermentation efficiency.

#### Digestibility of DM


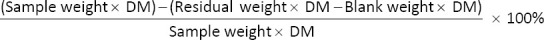


#### Digestibility of OM


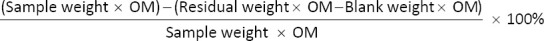


#### Total VFA content

The VFA production was measured by steam distillation using the General Laboratory Procedure method [22].

VFA = (a − b) × Hydrogen chloride (HCl)(1000/5) mM

Description: (a) Titration volume of 5 mL of sodium hydroxide blank; (b) sample titration volume

#### NH_3_

NH_3_ production from rumen fluid was measured as described by Conway and O’Malley [23]. The supernatant resulting from 4 h of incubation was centrifuged at 1,372 × *g* for 15 min. Subsequently, 1 mL was placed in the Conway glass, followed by 1 mL of a 0.005 M saturated sodium carbonate (Na_2_CO_3_) solution, and 1 mL of boric acid was added to the middle Conway glass. Conway’s glass, which has a lid smeared with Vaseline, is tightly closed to make it airtight. The supernatant and Na_2_CO_3_ were evenly mixed until the color changed from blue to red. After incubation for 24 h, boric acid was titrated with 0.005 M sulfuric acid. The NH3 concentration was calculated using the following formula:

NH_3_ = mL titration × N H_2_SO_4_ × 17 × 100 (mg/100 ml)

### Tannin analysis

Tannin content was determined using the Folin–Denis colorimetric method as described by AOAC [24]. The Folin–Denis reagent was prepared by combining 100 g sodium tungstate, 20 g phosphomolybdic acid, and 50 mL of 85% phosphoric acid in 750 mL of distilled water. The mixture was refluxed for 3 h, cooled, and distilled to a final volume of 1 L. A saturated Na_2_CO_3_ solution was prepared by dissolving 35 g anhydrous Na_2_CO_3_ in 100 mL of distilled water at 70°C–80°C, cooled, and stored overnight. A 100 ppm tannic acid standard was freshly prepared for each assay by dissolving 100 mg tannic acid in 1 L of distilled water.

For the standard curve, 1 mL–5 mL aliquots of the tannic acid solution were pipetted into 100 mL volumetric flasks containing 50 mL–70 mL distilled water and 2 mL Folin–Denis reagent, followed by 5 mL saturated Na_2_CO_3_. The mixtures were diluted to volume, mixed thoroughly, and allowed to stand for 40 min. Absorbance was measured at 725 nm using a spectrophotometer.

For sample preparation, 2 g of homogenized seaweed powder was mixed with 350 mL of distilled water, refluxed for 3 h, cooled, and filtered into a 500 mL volumetric flask, then made up to volume. A 2 mL aliquot of the filtrate was reacted with 2 mL Folin–Denis reagent and 5 mL Na_2_CO_3_ solution, diluted to volume, and left to stand for 40 min before measuring absorbance at 725 nm. Tannin concentration was expressed as tannic acid equivalent (mg/g DM).

### Saponin analysis

Saponin concentration was analyzed following a thin-layer chromatography (TLC) method. Approximately 1 g–2.5 g of sample was placed in a 25 mL volumetric flask, filled one-quarter with distilled water, and shaken mechanically for 2 h. The volume was then adjusted to the mark with distilled water, and the solution was left to stand for 24 h before filtration.

From the filtrate, 5 μL was spotted on an aluminum-backed Silica Gel 60 GF_254_ TLC plate, alongside 5 μL of a 190 ppm saponin standard. The plate was developed in a chloroform: ethanol (49:1) mobile phase containing a few drops of ethyl acetate, until the solvent front reached approximately 15 cm. After air drying, the developed plate was scanned using a Camag 3 TLC scanner at 292 nm for quantification.

### Amino acid analysis

Amino acid composition was determined using high-performance liquid chromatography (HPLC) (Agilent 1220 Infinity II system). Approximately 0.1 g of sample was hydrolyzed in 5 mL of 6 N HCl for 22 h at 112°C, then diluted to 25 mL with deionized water. The solution was filtered through a 0.22 μm PTFE syringe filter into a 1.5 mL vial.

For derivatization, 10 μL of the filtrate was mixed with 70 μL borate buffer and 20 μL derivatization reagent, reacted for 10 min at 55°C, and injected into the HPLC system. Individual amino acids were identified and quantified based on retention times compared to authentic standards [25].

### Fatty acid analysis

Fatty acids were analyzed through gas chromatography–flame ionization detection following methyl ester derivatization. After *in vitro* incubation, fermentation was terminated by adding 500 μL of 2% mercuric chloride (w/v). Samples were transferred to 100 mL flasks, frozen at −60°C, and freeze-dried for 48 h. About 50 mg of the dried sample was weighed into a screw-cap tube and methylated using 2148 μL methanol, 990 μL toluene, 66 μL 99.9% sulfuric acid, 1000 μL dimethyl sulfoxide, and 2 mL hexane. The mixture was heated at 80°C for 2 h, cooled, and the hexane layer was collected and evaporated under nitrogen. The residue was re-dissolved in 500 μL dichloromethane, and 250 μL was injected into a Shimadzu GC-2014 equipped with a Rt-2560 capillary column (100 m × 0.25 mm, 0.2 μm film thickness). Helium was used as the carrier gas at 1.12 mL/min. Fatty acid methyl esters (FAMEs) were identified by comparing retention times with those of the Supelco 37 Component FAME Mix standard.

### Metabolomic analysis (bioactive compounds)

Bioactive compound profiling was performed using untargeted metabolomics through liquid chromatography–high-resolution mass spectrometry [26]. Analyses were conducted on a Thermo Scientific Vanquish ultrahigh-performance liquid chromatography system (Thermo Fisher, USA) coupled with a Q Exactive Hybrid Quadrupole-Orbitrap (Thermo Fisher Scientific) mass spectrometer. Separation was achieved using a Phenyl-Hexyl analytical column (100 × 2.1 mm, 2.6 μm) at 40°C, with a mobile phase of (A) water + 0.1% formic acid and (B) methanol + 0.1% formic acid, at a flow rate of 0.3 mL/min. The gradient began at 5% B, ramped to 90% over 16 min, held for 4 min, and returned to initial conditions by 25 min.

Full-scan and data-dependent MS^2^ acquisition were performed in both positive and negative ionization modes, with nitrogen as the carrier (32 AU), auxiliary (8 AU), and sweep gas (4 AU). The spray voltage was 3.30 kV, capillary temperature was 320°C, and heater temperature was 30°C. The scan range was m/z 66.7–1000, at a resolution of 70,000 (MS^1^) and 17,500 (MS^2^). Instrument control and data acquisition were managed using XCalibur 4.4 software (Thermo Scientific). Weekly calibration using Pierce ESI ion solutions ensured mass accuracy (<5 ppm) and system stability.

### Statistical analysis

All experimental data were statistically analyzed using IBM Statistical Package for the Social Sciences Statistics version 24.0 (IBM Corp., Armonk, NY, USA) [27]. Differences between seaweed species were evaluated through one-way analysis of variance. When significant effects were detected, Duncan’s Multiple Range Test was applied for *post hoc* mean comparison. Results were considered statistically significant at p < 0.05, and data were expressed as mean ± standard deviation.

## RESULTS

### Chemical composition of seaweed species

The proximate and mineral compositions of *S. binderi* (brown seaweed) and *K. striatum* (green seaweed) are summarized in Table 1. The CP content ranged from 6.84% to 7.52%, with green seaweed exhibiting a higher CP level (7.52%) than brown seaweed (6.84%). Among macrominerals, green seaweed contained higher levels of calcium (0.41%), sodium (0.15%), and magnesium (0.73%), whereas brown seaweed exhibited greater concentrations of microminerals such as iron (4.07 ppm), manganese (0.86 ppm), and zinc (10.54 ppm). These variations reflect species-specific mineral accumulation patterns and suggest that both seaweeds can serve as potential mineral supplements for ruminant feed formulations.

**Table 1:** Chemical composition of seaweed species (all values are based on dry matter).

Composition	Brown seaweed (*Sargassum binderi*)	Green seaweed (*Kappaphycus striatum*)
Nutrients (%)		
CP	6.84	7.52
CF	1.91	0.64
Ash	17.94	18.46
Tannin	0.50	0.37
Saponin	0.97	0.85
Macro minerals (%)		
Ca	0.33	0.41
P	0.28	0.28
Na	0.14	0.15
Mg	0.64	0.73
S	0.03	0.03
NaCl	4.62	3.68
Micro minerals (ppm)		
Fe	4.07	3.73
Mn	1.00	0.86
Zn	10.54	8.64
Cu	9.61	9.98

CP = Crude protein, CF = Crude fiber, Ca = Calcium, P = Phosphorus, Na = Natrium, Mg = Magnesium, S = Sulfur, NaCl = Sodium chloride, Fe = Iron, Mn = Manganese, Zn = Zinc, Cu = Copper.

### *In vitro* digestibility of DM and OM

The *in vitro* DMD and OMD values are presented in Table 2. Both parameters are critical indicators of feed quality and energy availability in ruminant nutrition. Significant differences (p < 0.01) were observed between the two seaweed species. Green seaweed demonstrated superior digestibility with DMD and OMD values of 73.56% and 72.71%, respectively, compared with 46.38% and 44.99% for brown seaweed. These results suggest that *K. striatum* possesses a more favorable nutrient composition and degradability profile, likely due to its lower crude fiber and polysaccharide content compared with *S. binderi*.

**Table 2:** *In vitro* digestibility of dry and organic seaweed matter.

Digestibility	Brown seaweed (*Sargassum binderi*)	Green seaweed (*Kappaphycus striatum*)	p-value
DMD (%)	46.38^a^ ± 5.37	73.56^b^ ± 5.89	0.002
OMD (%)	44.99^a^ ± 5.52	72.71^b^ ± 6.08	0.001

^a,b^Superscripts that different each row are highly significant difference (p < 0.01). DMD = Dry matter digestibility, OMD = Organic matter digestibility.

### Rumen fermentation characteristics (pH, NH_3_, and total VFA)

The rumen fermentation parameters of the two seaweed species are summarized in Table 3. The ruminal pH remained stable across treatments (7.00–7.21), showing no significant differences (p > 0.05), and remained within the optimal physiological range (6.2–7.2) for microbial activity. However, both NH_3_ and total VFA concentrations varied significantly (p < 0.01) between species. Brown seaweed produced a higher VFA concentration (151.75 mM) than green seaweed (136.75 mM), indicating enhanced carbohydrate fermentation potential, whereas green seaweed exhibited higher NH_3_ levels (26.78 mM) than brown seaweed (22.21 mM), suggesting improved nitrogen utilization efficiency and microbial protein synthesis.

**Table 3:** Rumen fermentation characteristics (pH, NH_3_, and total VFA) of the seaweed species.

Characteristics	Brown seaweed (*Sargassum binderi*)	Green seaweed (*Kappaphycus striatum*)	p-value
pH	7.21 ± 0.25	7.00 ± 0.03	0.321
Total VFA (mM)	151.75^b^ ± 2.36	136.75^a^ ± 2.36	0.001
NH_3_ production (mM)	22.21^a^ ± 0.41	26.78^b^ ± 0.60	0.001

^a,b^Superscripts that differ for each row are highly significant difference (p < 0.01). NH_3_ = Ammonia, VFAs = Volatile fatty acids.

### Amino acid composition of seaweed species

The amino acid profiles of *S. binderi* and *K. striatum* (Table 4) revealed the presence of both essential (threonine, valine, methionine, lysine, isoleucine, leucine, and phenylalanine) and non-essential (aspartic acid, serine, glutamic acid, glycine, arginine, alanine, proline, cystine, and tyrosine) amino acids. Green seaweed exhibited higher concentrations of most essential amino acids, particularly valine, leucine, and lysine, suggesting greater potential for supporting growth and microbial protein synthesis in ruminants. The balanced amino acid composition in both seaweeds indicates their suitability as alternative protein sources or supplementary ingredients in ruminant diets.

**Table 4:** Amino acid composition of seaweed species.

Amino acid	Brown seaweed (*Sargassum binderi*)	Green seaweed (*Kappaphycus striatum*)
Essential amino acid content (%)		
Histidine	0.14 ± 0.01	0.06 ± 0.00
Threonine	0.45 ± 0.01	0.78 ± 0.04
Valine	0.67 ± 0.01	1.46 ± 0.02
Methionine	0.13 ± 0.01	0.04 ± 0.00
Lysine	0.46 ± 0.01	0.72 ± 0.00
Iso-leucine	0.46 ± 0.00	0.93 ± 0.00
Leucine	0.65 ± 0.00	1.10 ± 0.01
Phenylalanine	0.49 ± 0.01	0.68 ± 0.01
Non-essential amino acids (%)		
Aspartic acid	0.86 ± 0.00	1.83 ± 0.01
Serine	0.37 ± 0.01	0.78 ± 0.00
Glutamic acid	1.15 ± 0.01	1.89 ± 0.03
Glycine	0.46 ± 0.00	0.96 ± 0.03
Arginine	0.42 ± 0.02	0.48 ± 0.01
Alanine	0.42 ± 0.01	1.82 ± 0.03
Proline	0.30 ± 0.00	0.54 ± 0.04
Cystine	0.00 ± 0.00	0.08 ± 0.01
Tyrosine	0.23 ± 0.00	0.20 ± 0.01

### Fatty acid composition of seaweed species

The fatty acid composition of brown and green seaweeds (Table 5) demonstrated substantial variation among saturated fatty acid (SFA), monounsaturated fatty acid (MUFA), and polyunsaturated fatty acid (PUFA). Both species contained high levels of SFAs, notably palmitic acid (C16:0) and stearic acid (C18:0). Green seaweed exhibited slightly higher PUFA content, including linoleic acid (C18:2), α-linolenic acid (C18:3), and conjugated linoleic acid (CLA), which are known to modulate rumen fermentation, improve milk fatty acid profiles, and contribute to methane reduction. These findings suggest that tropical seaweeds can be a valuable source of lipids for enhancing the nutritional and functional quality of ruminant feed.

**Table 5:** Fatty acid composition of seaweed species.

Fatty acid	Brown seaweed (*Sargassum binderi*)	Green seaweed (*Kappaphycus striatum*)
Saturated fatty acids (%)		
C4:0	0.34 ± 0.08	0.34 ± 0.04
C6:0	0.38 ± 0.08	0.40 ± 0.25
C8:0	0.46 ± 0.07	0.31 ± 0.15
C10:0	0.47 ± 0.20	0.81 ± 0.76
C11:0	0.54 ± 0.18	0.70 ± 0.41
C12:0	1.53 ± 2.11	2.92 ± 0.49
C13:0	0.42 ± 0.12	0.34 ± 0.22
C14:0	2.23 ± 0.46	3.04 ± 1.09
C15:0	0.67 ± 0.23	0.53 ± 0.20
C16:0	35.51 ± 3.24	22.98 ± 5.64
C17:0	0.46 ± 0.14	0.35 ± 0.12
C18:0	38.03 ± 10.02	47.56 ± 6.47
C20:0	1.49 ± 0.44	1.23 ± 0.27
C21:0	0.50 ± 0.08	0.44 ± 0.17
C22:0	0.69 ± 0.14	0.63 ± 0.06
C23:0	0.44 ± 0.08	0.45 ± 0.22
C24:0	0.81 ± 0.25	0.70 ± 0.13
C14:1	0.91 ± 0.15	0.99 ± 0.26
C15:1	0.46 ± 0.11	0.51 ± 0.33
C16:1	0.77 ± 0.11	1.03 ± 0.15
C17:1	0.48 ± 0.14	0.38 ± 0.07
C18:1 trans	1.64 ± 1.27	1.78 ± 0.29
C18:1 cis	0.84 ± 0.32	0.91 ± 0.24
C20:1	0.77 ± 0.82	1.54 ± 0.38
C22:1n9	0.77 ± 0.34	0.97 ± 0.33
C24:1	0.62 ± 0.29	0.77 ± 0.15
Polyunsaturated fatty acids (%)		
C18:2 trans	0.44 ± 0.12	0.37 ± 0.02
C18:2 cis	0.46 ± 0.16	0.34 ± 0.12
C18:3n6	0.53 ± 0.21	0.43 ± 0.13
C18:3n3	0.36 ± 0.06	0.35 ± 0.09
C20:2	0.51 ± 0.20	0.42 ± 0.14
C20:3n6	0.44 ± 0.06	0.39 ± 0.12
C20:3n3	0.60 ± 0.30	0.57 ± 0.33
C20:4n6	0.59 ± 0.40	0.66 ± 0.18
C22:2n6	0.46 ± 0.09	0.35 ± 0.13
C20:5n3	0.45 ± 0.08	0.42 ± 0.16
C22:6	0.47 ± 0.12	0.38 ± 0.10
CLA	2.47 ± 1.38	2.71 ± 1.51

### Bioactive compounds in seaweed

Metabolomic profiling (Table 6) identified approximately 85 bioactive compounds across both seaweed species, highlighting notable biochemical diversity. Brown seaweed (*S. binderi*) contained higher levels of lipid-derived bioactives such as 1-stearoylglycerol, L-α-palmitin, and arachidonic acid (AA), which are associated with enhanced fat metabolism and potential antimicrobial properties. In contrast, green seaweed (*K. striatum*) exhibited unique metabolites, including betaine, cholecalciferol (vitamin D_3_), and 2-amino-1,3,4-octadecanetriol, which contribute to osmoregulation, nutrient absorption, and microbial modulation in the rumen.

**Table 6:** Metabolomics analysis of bioactive compounds of seaweed species.

No.	Retention time (min)	Error (%) (ppm)	Mass (m/z)	Formula	Name	Area (max.)	Percentage Relative	

							Brown seaweed *(Sargassum binderi)*	Green seaweed *(Kappaphycus striatum)*
1	14.433	−1.08	238.22941	C_16_H_30_O	( ± )-Muscone	390015964.4	-	1.04
2	8.762	−0.3	188.12006	C_13_H_16_O	(2E)-5-methyl-2-phenylhex-2-enal	9451044.406	0.21	-
3	17.145	−0.51	373.33428	C_25_H_43_NO	(2E,4E,16Z)-1-(1-Piperidinyl)-2,4,16-icosatrien-1-one	17285439.26	0.61	0.36
4	16.267	−0.67	345.30293	C_23_H_39_NO	(2E,4Z,12E)-1-(1-Piperidinyl)-2,4,12-octadecatrien-1-one	37449507.65	1.40	0.78
5	17.259	−0.04	610.4597	C_39_H_62_O_5_	(2S)-1-Hydroxy-3-[(9Z)-9-tetradecenoyloxy]-2-propanyl (4Z,7Z,10Z,13Z,16Z,19Z)-4,7,10,13,16,19-docosahexaenoate	10769153.81	0.23	-
6	0.863	−0.48	175.12076	C_8_H_17_NO_3_	(2S)-2-Amino-8-hydroxyoctanoic acid	12506090.44	-	0.26
7	10.871	−0.31	234.12552	C_14_H_18_O_3_	(2Z)-2-(3-Hydroxybenzylidene) HCA	7575394.515	-	0.16
8	16.711	−0.85	428.36507	C_29_H_48_O_2_	(3b,24R,24′R)-fucosterol epoxide	18271916.4	1.54	-
9	16.822	−0.88	323.31853	C_21_H_41_NO	1-(14-methylhexadecanoyl) pyrrolidine	20344559.8	0.41	0.42
10	16.471	−0.54	359.31862	C_24_H_41_NO	1-(6-Nonyl-3-pyridinyl)-1-decanone	9049297.946	0.28	0.19
11	18.24	−0.53	590.49071	C_37_H_66_O_5_	1-(9Z-hexadecenoyl)-2-(9Z,12Z-octadecadienoyl)-sn-glycerol	15800114.76	0.34	-
12	19.051	−0.73	594.52189	C_37_H_70_O_5_	1-[(11Z)-octadecenoyl]-2-hexadecanoyl-sn-glycerol	30608546.69	0.67	
13	13.372	−0.14	300.2664	C_18_H_36_O_3_	12-HSA	63775798.39	-	0.57
14	18.37	0.45	566.49128	C_35_H_66_O_5_	1-oleoyl-2-myristoyl-sn-glycerol	23664426.41	0.52	-
15	15.498	−1.41	358.3078	C_21_H_42_O_4_	1-Stearoylglycerol	526188303.3	13.19	10.94
16	9.059	0.13	157.14668	C_9_H_19_NO	2,2,6,6-Tetramethyl-1-piperidinol (TEMPO)	114066381.7	2.55	2.37
17	15.829	0.32	340.24034	C_23_H_32_O_2_	2,2’- Methylenebis (4-methyl-6-tert-butylphenol)	17676410.97	0.17	0.37
18	10.159	−1.85	317.29241	C_18_H_39_NO_3_	2-Amino-1,3,4-octadecanetriol	344292671.1	-	7.16
19	14.338	0.31	378.27713	C_23_H_38_O_4_	2-Arachidonoyl glycerol	39729855.18	0.87	-
20	0.922	0.71	164.06859	C_6_H_12_O_5_	2-Deoxyhexopyranose	68424042.95	1.49	-
21	10.606	−0.33	160.08876	C_11_H_12_O	2-Phenyl-4-pentenal	7118691.627	-	0.15
22	15.493	−0.32	266.26088	C_18_H_34_O	2-Tetradecylcyclobutanone	36688220.58	0.87	0.76
23	12.896	0.01	402.29814	C_22_H_42_O_6_	3,6-Anhydro-1-O-palmitoylhexitol	10188630.88	0.22	-
24	9.157	−0.21	180.11499	C_11_H_16_O_2_	3-BHA	49110954.99	1.44	1.02
25	17.757	−0.05	434.33959	C_27_H_46_O_4_	3a,7α-dihydroxy-5b-cholestan-26-oic acid	9408718.688	0.21	-
26	18.037	−1.36	382.32305	C_27_H_42_O	4,6-CHOLESTADIEN-3-ONE	57622639.78	-	1.20
27	0.883	−0.31	158.06909	C_6_H_10_N_2_O_3_	4-Methyleneglutamine	44642195.78	-	0.93
28	15.898	−0.63	400.33388	C_27_H_44_O_2_	7-OXOCHOLESTEROL	165546739.4	-	4.04
29	0.817	−1.06	145.11012	C_7_H_15_NO_2_	Acetylcholine	72456417.29	-	1.51
30	0.992	−0.37	135.05445	C_5_H_5_N_5_	Adenine	40818196.12	-	-
31	1.118	0.59	267.09691	C_10_H_13_N_5_O_4_	Adenosine	12289335.78	0.27	-
32	0.947	0.56	146.05799	C6H10 O4	Adipic acid	63462858.94	1.38	-
33	12.517	0.02	267.21984	C16 H29 N O_2_	Allopumiliotoxin 267A	8416470.887	0.20	0.17
34	15.141	−0.57	304.24006	C20 H32 O_2_	Arachidonic acid	166403511.8	3.41	-
35	0.836	0.87	117.07908	C5 H11 N O_2_	Betaine	1008655865	-	7.49
36	0.898	−0.46	163.08438	C_6_H_13_NO_4_	Bicine	23804493.3	-	0.49
37	11.533	−0.48	414.20404	C_24_H_30_O_6_	Bis (4-ethylbenzylidene) sorbitol	272017976.3	1.24	5.65
38	10.583	−0.82	386.17262	C_22_H_26_O_6_	Bis (methylbenzylidene) sorbitol	82132626.97	1.49	2.43
39	17.544	0.48	511.49669	C_32_H_65_NO_3_	C14-Dihydroceramide	96434175.56	1.33	2.00
40	14.865	0.01	269.27187	C_17_H_35_NO	Capsi-amide	29610543.73	0.84	0.62
41	14.429	−2.06	384.33843	C_27_H_44_O	Cholecalciferol	27835094.5	-	4.04
42	18.376	−1.18	384.33876	C_27_H_44_O	Cholest-4-en-3-one	22074204.86	-	0.46
43	10.647	−0.19	239.13097	C_16_H_17_NO	Diphenamid	6641616.34	0.27	0.14
44	0.845	−0.65	161.10509	C_7_H_15_NO_3_	DL-Carnitine	29509263.43	0.68	0.61
45	0.868	0.41	131.09468	C_6_H_13_NO_2_	DL-b-Leucine	21188763.39	-	0.44
46	10.1	−0.04	185.21434	C_12_H_27_N	Dodecylamine	9073936.808	0.19	0.19
47	15.044	−0.84	352.26106	C_21_H_36_O_4_	Doxaprost	9596709.535	-	0.20
48	17.183	−0.09	412.33409	C_28_H_44_O_2_	Doxercalciferol	34487323.69	0.75	-
49	10.603	0.86	294.18336	C_17_H_26_O_4_	Embelin	66457570.65		1.38
50	14.57	0.53	282.25603	C_18_H_34_O_2_	Ethyl palmitoleate	18654034.17	0.94	0.39
51	18.621	0.17	410.35494	C_29_H_46_O	FF-MAS	27291772.73	0.60	-
52	0.919	1.51	128.04754	C_6_H_8_O_3_	Furaneol	50254381.09	1.10	-
53	10.957	−0.07	465.30901	C_26_H_43_NO_6_	Glycocholic acid	16468790.58	046	0.29
54	14.433	0.23	255.25627	C_16_H_33_NO	Hexadecanamide	294500168.6	0.50	-
55	10.301	−0.85	405.34508	C_22_H_47_NO_5_	Hydrolyzed fumonisin B1	8439053.167	-	0.18
56	11.534	0.89	118.07836	C_9_H_10_	Indane	10010312.16	-	0.21
57	6.886	−0.22	152.0837	C_9_H_12_O_2_	Isopropyl catechol	8444136.746	0.18	-
58	11.866	−0.5	199.19351	C_12_H_25_NO	Lauramine	13811316.3	0.25	029
59	1.175	0.53	131.0947	C_6_H_13_NO_2_	L-Norleucine	12734399.39	0.28	-
60	1.5	0.2	165.07901	C_9_H_11_NO_2_	L-Phenylalanine	98530133.26	0.39	-
61	14.445	−1.7	330.27645	C_19_H_38_O_4_	L-α-PALMITIN	726894685.1	19.51	15.11
62	16.346	−0.29	308.27144	C_20_H_36_O_2_	Mandenol	6729860.986	-	0.14
63	14.84	−2.08	356.29192	C_21_H_40_O_4_	Monoolein	7709789.646	0.37	-
64	10.143	−1.12	287.24572	C_16_H_33_NO_3_	N, N-bis (2-hydroxyethyl) dodecanamide	173594999.4	2.30	3.61
65	15.804	−0.97	255.25597	C_16_H_33_NO	N, N-Diethyldodecanamide	143068219.5	1.66	-
66	2.06	0.48	129.15181	C_8_H_19_N	N, N-Diisopropylethylamine (DIPEA)	41032606.51	2.64	2.24
67	8.647	−0.93	201.20908	C_12_H_27_NO	N, N-Dimethyldecylamine N-oxide	5483620.532	-	0.11
68	0.832	−1.23	188.11586	C_8_H_16_N_2_O_3_	N6-Acetyllysine	14510190.22	-	0.30
69	4.074	−0.47	179.09455	C_10_H_13_NO_2_	N-Acetyltyramine	14894415.91	-	-
70	10.561	−0.36	225.11528	C_15_H_15_NO	Aenone A	49652356.13	1.88	1.03
71	6.006	−0.55	241.14653	C_16_H_19_NO	N-Desmethyldiphenhydramine	143563949.7	3.61	2.98
72	1.108	1.07	122.04814	C_6_H_6_N_2_O	Nicotinamide	11169438.16	0.24	-
73	2.85	−1.71	226.16774	C_12_H_22_N_2_O_2_	Nylon cyclic dimer	29565916.33	-	0.47
74	15.287	−0.05	256.24022	C_16_H_32_O_2_	Palmitic acid	6529318.967	-	0.14
75	0.85	−0.26	142.07419	C_6_H_10_N_2_O_2_	Piracetam	147963746.2	-	2.19
76	11.243	−0.7	301.29787	C_18_H_39_NO_2_	Sphinganine	15328496.76	0.83	0.45
77	15.292	−0.51	283.28737	C_18_H_37_NO	Stearamide	16648925.92	13.71	7.62
78	10.134	−3.61	276.20793	C_18_H_28_O_2_	Stearidonic acid	14949127.24	0.77	-
79	10.836	0.07	421.28285	C_24_H_39_NO_5_	Talatizamine	16408537.5	0.45	-
80	15.968	−0.26	297.30309	C_19_H_39_NO	Tridemorph	12250835.12	0.52	0.25
81	0.881	1.72	117.07918	C_5_H_11_NO_2_	Valine	19875324.09	0.43	-
82	5.671	−0.73	196.1098	C_11_H_16_O_3_	ZINGEROL	27999110.3	5.61	1.48
83	12.653	−0.05	278.22457	C_18_H_30_O_2_	α-Eleostearic acid	18057121.86	0.39	-
84	14.538	−1.05	278.22429	C_18_H_30_O_2_	α-Linolenic acid	147691616	0.67	-

The distinct bioactive profiles suggest that these seaweeds possess species-specific functional properties, offering dual benefits as nutritional enhancers and methane-mitigating feed additives in tropical ruminant production systems.

## DISCUSSION

### Chemical composition of seaweed species

Seaweeds are recognized as nutrient-dense marine resources rich in proteins, carbohydrates, amino acids, lipids, vitamins, and minerals [5]. Among different seaweed groups, green seaweeds generally exhibit higher protein content than brown species, while red seaweeds often surpass both, with CP levels ranging from 18% to 38% [3]. However, the protein content of seaweed is highly variable and influenced by species differences, environmental conditions, and seasonal factors [28, 29].

The mineral composition of seaweeds, often exceeding 20%, highlights their potential as significant mineral sources for ruminants, either as feed supplements or as partial forage substitutes [30]. Seaweeds can contain mineral concentrations 10–100 times higher than terrestrial plants and vegetables [31]. In our study, both brown seaweed (*S. binderi*) and green seaweed (*K. striatum*) exhibited high levels of macro- (Ca, P, Na, Mg, and S) and micro-minerals (Fe, Mn, Zn, and Cu), fulfilling the nutritional requirements of ruminants and reinforcing their role as organic mineral supplements in livestock feeding systems, which is consistent with the study of Kustantinah *et al*. [15].

### *In vitro* digestibility of DM and OM

The *in vitro* DMD and OMD differed markedly between seaweed types. Green seaweed demonstrated the highest DMD (73.56%) and OMD (72.71%), while brown seaweed exhibited significantly lower digestibility values (DMD 46.38% and OMD 44.99%). These variations are largely attributed to differences in crude fiber structure, polysaccharide composition, and anti-nutritional compounds.

Green seaweed’s superior digestibility may be associated with its less complex tissue matrix and lower levels of polysaccharides (such as fucoidan and alginate), which are abundant in brown seaweeds and limit microbial degradation. Conversely, brown seaweeds possess rigid cell walls that are more resistant to enzymatic breakdown in the rumen. Previous findings by Aslamyah *et al*. [32] suggest that fermented or processed seaweeds can enhance digestibility and overall rumen efficiency. Hence, incorporating tropical seaweeds into ruminant diets may improve feed utilization and reduce environmental impact through enhanced nutrient conversion and reduced methane losses.

### Rumen fermentation characteristics: pH, NH_3_, and total VFA

Rumen pH is a vital determinant of microbial activity, fermentation efficiency, and overall rumen health. In our study, green seaweed recorded a pH of 7.00, while brown seaweed exhibited 7.21, both within the optimal range (6.2–7.2) for maintaining microbial stability and efficient fermentation. This equilibrium between acidic VFAs and alkaline NH_3_ maintains a balanced rumen environment conducive to microbial growth [32–36].

VFAs, the principal energy source for ruminants, reflect the extent of carbohydrate fermentation [37]. Brown seaweed yielded the highest total VFA concentration (151.75 mM), followed by green seaweed (136.75 mM), both within the typical physiological range (70–150 mM) [37]. High VFA concentrations in the rumen indicate optimal energy provision for livestock and carbon skeletons for rumen microbes [38]. Various factors influence VFA concentration including the digestibility, type and quality of feed fermented by rumen microbes [39–41].

The NH_3_ concentration was highest in green seaweed (26.78 mM) and lowest in brown seaweed (22.21 mM). Although these values slightly exceed the optimal range (6–21 mM) [42], they indicate efficient protein degradation and ammonium assimilation by rumen microbes. The higher NH_3_ concentration in *K. striatum* may result from its enhanced surface area-to-volume ratio and greater nitrogen content, while *S. binderi* exhibits a higher C: N ratio, which can limit NH_3_ release and subsequent microbial protein synthesis [43, 44].

### Amino acid composition of seaweed species

The amino acid profiles of the studied seaweeds revealed that green seaweed (*K. striatum*) contained higher levels of essential amino acids such as valine (1.46%), leucine (1.10%), and lysine (0.72%) compared with brown seaweed (*S. binderi*). These findings align with previous studies by Gaillard *et al*. [45] and Pirian *et al*. [28], indicating that seaweeds’ protein and amino acid contents vary with species and harvest season. The amino acid composition of many seaweeds is comparable to soybean meal, highlighting their potential as alternative protein sources for livestock [5].

Lysine, in particular, is crucial for milk protein synthesis in dairy cattle and has been associated with increased milk yield and improved animal health [46]. Although *S. binderi* contained lower essential amino acid levels, both seaweeds provided nutritionally balanced amino acid profiles suitable for rumen microbial metabolism and animal growth.

*K. striatum* also showed high levels of non-essential amino acids, including glutamic acid (1.89%), alanine (1.87%), and aspartic acid (1.83%), which play vital roles in fiber digestion and microbial activity [47]. Similarly, *S. binderi* provided moderate concentrations of glutamic acid (1.15%) and aspartic acid (0.86%), which support microbial proliferation and rumen fermentation. Notably, certain amino acids may also contribute to methane reduction by suppressing methanogenic archaea during rumen fermentation [47].

### Fatty acid composition of seaweed species

Seaweeds are rich in PUFAs, and their specific fatty acid profiles differ across species and environmental conditions [5]. The balance between SFA and USFA, including MUFA and PUFA fractions, affects rumen fermentation and lipid metabolism [3].

Both *S. binderi* and *K. striatum* contained abundant palmitic acid (C16:0) and stearic acid (C18:0), the predominant SFAs. *K. striatum* had higher stearic acid (47.56%), while *S. binderi* showed greater palmitic acid (35.51%), influencing milk fat composition and saturated fat content [48]. Short-chain fatty acids (e.g., butyric acid and caproic acid) were also detected, contributing to rapid energy metabolism [49].

The MUFA fraction, including C18:1 trans, was highest in *K. striatum* (1.78%) and *S. binderi* (1.64%). Elevated MUFA levels are associated with improved milk fatty acid profiles, reduced methane emissions, and inhibited rumen biohydrogenation [50]. The presence of longer-chain MUFAs such as C20:1 and C22:1n9 in green seaweed further enhances its potential as a functional feed additive.

Both seaweeds contained bioactive PUFAs such as linoleic acid (C18:2), α-linolenic acid (C18:3), eicosapentaenoic acid (EPA, C20:5n3), docosahexaenoic acid (DHA, C22:6), and CLA. Notably, *K. striatum* exhibited the highest CLA (2.71%), while *S. binderi* had 2.47%, highlighting their potential to enhance milk PUFA content and reduce enteric methane formation. The presence of omega-3 and omega-6 fatty acids further supports immune modulation, meat quality, and animal health [3].

### Bioactive compounds in seaweed

Seaweeds contain diverse bioactive metabolites, making them promising functional feed ingredients for improving rumen efficiency and animal health [51]. Metabolomic profiling (Table 5) identified approximately 80 distinct bioactive compounds, influenced by environmental conditions, light exposure, and nutrient availability, which regulate secondary metabolite biosynthesis [51]. Although bromoform, a known anti-methanogenic compound, was absent, consistent with its confinement to certain red seaweeds [52], numerous other beneficial bioactives were identified.

In brown seaweed (*S. binderi*), predominant lipid-derived bioactives such as L-α-palmitin (19.51%), 1-stearoylglycerol (13.19%), and stearamide (13.71%) serve as energy-dense molecules with potential antimicrobial effects in the rumen [53]. The presence of AA, 3.41%, a prostaglandin precursor, suggests a role in immune regulation and reproductive performance.

Conversely, green seaweed (*K. striatum*) contained unique metabolites, including betaine (7.49%), a natural osmoprotectant that aids in heat stress tolerance and cellular osmotic balance; cholecalciferol (vitamin D_3_ 4.04%), which supports calcium–phosphorus metabolism and bone integrity; and 2-amino-1,3,4-octadecanetriol (7.16%), an amino alcohol potentially involved in microbial protein synthesis and fiber digestion [54].

The contrasting bioactive profiles suggest species-specific functional advantages: *S. binderi* is better suited as an energy-rich supplement for lactating or fattening ruminants, while *K. striatum* serves as a functional feed additive that enhances stress resilience and nutrient absorption. Nevertheless, *in vivo* validation is essential to confirm optimal inclusion rates, assess long-term safety, and ensure that high dosages do not disrupt rumen fermentation dynamics.

## CONCLUSION

This study comprehensively evaluated the nutritional composition, *in vitro* digestibility, rumen fermentation characteristics, amino acid and fatty acid profiles, and metabolomic bioactive compounds of two tropical seaweed species, brown seaweed (*S. binderi*) and green seaweed (*K. striatum*, to assess their potential as functional feed additives for ruminants. The findings revealed notable interspecies variation, highlighting distinct nutritional and functional properties relevant to tropical livestock feeding systems.

Green seaweed (*K. striatum*) exhibited higher CP (7.52%), DMD (73.56%), and OMD (72.71%) than brown seaweed (*S. binderi*), which recorded lower digestibility values (46.38% and 44.99%, respectively). Significant differences (p < 0.01) were observed in NH_3_ and VFA production, with *K. striatum* producing higher NH_3_ concentrations (26.78 mM), indicative of enhanced microbial protein synthesis, while *S. binderi* generated higher VFA levels (151.75 mM), suggesting superior carbohydrate fermentation efficiency. Both species contained balanced profiles of essential and non-essential amino acids and abundant PUFAs such as linoleic acid, α-linolenic acid, EPA, DHA, and CLA. Metabolomic analysis identified approximately 85 bioactive compounds, including lipid derivatives, vitamins, and osmolytes, underscoring their potential roles in modulating rumen fermentation and animal metabolism.

These findings demonstrate that tropical seaweeds can serve as sustainable, locally available, and nutritionally rich feed ingredients capable of enhancing rumen fermentation efficiency, improving nutrient digestibility, and contributing to methane emission mitigation. Specifically, *S. binderi* may serve as an energy-dense supplement for high-production stages such as lactation or fattening, whereas *K. striatum* may function as a functional additive that supports microbial balance, nutrient assimilation, and stress tolerance under tropical rearing conditions.

This study is among the first to present a comprehensive characterization of tropical seaweeds using integrated chemical, biochemical, and metabolomic analyses, providing a strong foundation for their strategic use in sustainable livestock feeding. The combination of nutritional, digestibility, and metabolomic data offers a holistic understanding of their feed potential beyond conventional proximate analysis. However, as an *in vitro* study, these findings reflect controlled rumen simulation rather than the complexity of *in vivo* conditions, where factors such as feed intake behavior, rumen kinetics, and long-term microbial adaptation can alter the outcomes. Additionally, the absence of bromoform or halogenated compounds, key antimethanogenic agents in some red seaweeds, suggests that methane mitigation from these tropical species may rely on alternative biochemical pathways.

Further research should focus on *in vivo* validation to determine optimal inclusion levels, evaluate animal performance, milk composition, methane emissions, and assess economic feasibility for smallholder farmers in tropical regions. Long-term feeding trials, coupled with microbial community profiling and metabolite flux analysis, will be essential to unravel the specific mechanisms by which these seaweeds influence rumen fermentation and GHG dynamics.

In conclusion, both *S. binderi* and *K. striatum* demonstrate significant promise as functional and sustainable feed additives for ruminants in tropical systems. Due to its rich nutritional profile, high digestibility, and unique bioactive compound content, this feed ingredient is a viable alternative to conventional feed. This substitution supports the global shift toward climate-smart and low-carbon livestock production. These findings contribute valuable baseline data to support the development of seaweed-based feeding strategies aimed at enhancing productivity, animal welfare, and environmental sustainability in tropical ruminant agriculture.

## AUTHORS’ CONTRIBUTIONS

LSS and MZ: Conceptualized and designed the study. FA, YM, and DD: Provided technical assistance and oversight. LSS, BVU, and SA: Conducted the laboratory observations, performed the data analysis, and drafted the manuscript. All authors have read and approved the final version of the manuscript.
